# Clinical experience with non‐invasive prenatal screening for single‐gene disorders

**DOI:** 10.1002/uog.23756

**Published:** 2022-01-05

**Authors:** P. Mohan, J. Lemoine, C. Trotter, I. Rakova, P. Billings, S. Peacock, C.‐Y. Kao, Y. Wang, F. Xia, C. M. Eng, P. Benn

**Affiliations:** ^1^ Natera, Inc., San Carlos CA USA; ^2^ Baylor Genetics Houston TX USA; ^3^ Baylor College of Medicine Houston TX USA; ^4^ Department of Genetics and Genome Sciences University of Connecticut Health Center Farmington CT USA

**Keywords:** autosomal dominant disorder, cell‐free DNA, next‐generation sequencing, non‐invasive prenatal testing, Noonan spectrum disorder, single‐gene disorder

## Abstract

**Objective:**

To assess the performance of a non‐invasive prenatal screening test (NIPT) for a panel of dominant single‐gene disorders (SGD) with a combined population incidence of 1 in 600.

**Methods:**

Cell‐free fetal DNA isolated from maternal plasma samples accessioned from 14 April 2017 to 27 November 2019 was analyzed by next‐generation sequencing, targeting 30 genes, to look for pathogenic or likely pathogenic variants implicated in 25 dominant conditions. The conditions included Noonan spectrum disorders, skeletal disorders, craniosynostosis syndromes, Cornelia de Lange syndrome, Alagille syndrome, tuberous sclerosis, epileptic encephalopathy, *SYNGAP1*‐related intellectual disability, CHARGE syndrome, Sotos syndrome and Rett syndrome. NIPT‐SGD was made available as a clinical service to women with a singleton pregnancy at ≥ 9 weeks' gestation, with testing on maternal and paternal genomic DNA to assist in interpretation. A minimum of 4.5% fetal fraction was required for test interpretation. Variants identified in the mother were deemed inconclusive with respect to fetal carrier status. Confirmatory prenatal or postnatal diagnostic testing was recommended for all screen‐positive patients and follow‐up information was requested. The screen‐positive rates with respect to the clinical indication for testing were evaluated.

**Results:**

A NIPT‐SGD result was available for 2208 women, of which 125 (5.7%) were positive. Elevated test‐positive rates were observed for referrals with a family history of a disorder on the panel (20/132 (15.2%)) or a primary indication of fetal long‐bone abnormality (60/178 (33.7%)), fetal craniofacial abnormality (6/21 (28.6%)), fetal lymphatic abnormality (20/150 (13.3%)) or major fetal cardiac defect (4/31 (12.9%)). For paternal age ≥ 40 years as a sole risk factor, the test‐positive rate was 2/912 (0.2%). Of the 125 positive cases, follow‐up information was available for 67 (53.6%), with none classified as false‐positive. No false‐negative cases were identified.

**Conclusions:**

NIPT can assist in the early detection of a set of SGD, particularly when either abnormal ultrasound findings or a family history is present. Additional clinical studies are needed to evaluate the optimal design of the gene panel, define target populations and assess patient acceptability. NIPT‐SGD offers a safe and early prenatal screening option. © 2021 The Authors. *Ultrasound in Obstetrics & Gynecology* published by John Wiley & Sons Ltd on behalf of International Society of Ultrasound in Obstetrics and Gynecology.

**CONTRIBUTION**

**What are the novel findings of this work?**

A total of 2208 women received a non‐invasive prenatal test result for a panel targeting 25 clinically significant single‐gene disorders, of whom 125 (5.7%) tested positive. High test‐positive rates were demonstrated for pregnancies with ultrasound abnormalities or with a family history of one of these disorders.

**What are the clinical implications of this work?**

Non‐invasive prenatal testing for single‐gene disorders has the potential to assist in the early detection of these disorders through cell‐free DNA analysis.

## Introduction

The presence of cell‐free fetal DNA in maternal plasma was described by Lo *et al*.^1^ in 1997. However, it is only in the last decade that molecular genetic technology has advanced sufficiently to allow clinical implementation of cell‐free fetal DNA‐based non‐invasive prenatal testing (NIPT), which is now commonly used in the detection of fetal chromosomal abnormalities^2^, ^3^, ^4^, ^5^. Originally targeting trisomy 21, 18 and 13, NIPT has expanded in scope to include sex‐chromosome aneuploidies^4^, ^6^ and a select group of microdeletions^7^.

Single‐gene disorders (SGD) are present in approximately 1% of births^8^. NIPT‐SGD is feasible for a broad range of monogenic disorders and is most straightforward when applied for the detection of dominant conditions with a high *de‐novo* rate or for paternally inherited dominant gene variants^9^, ^10^. A currently available NIPT‐SGD panel screens for 25 conditions that result from disease‐causing variants across 30 genes (Appendix S1), which have a combined incidence of 1 in 600 (0.17%)^11^. The conditions include Noonan spectrum disorders (NSD), skeletal disorders, craniosynostosis syndromes, Cornelia de Lange syndrome (CdLS), Alagille syndrome, tuberous sclerosis, epileptic encephalopathy, *SYNGAP1*‐related intellectual disability, CHARGE syndrome, Sotos syndrome and Rett syndrome. Although the testing can be considered as fully diagnostic for the conditions detected (i.e. non‐invasive prenatal diagnosis)^8^, we will refer to this testing as ‘screening’.

The clinical features associated with the disorders on the NIPT‐SGD panel can vary widely and, therefore, establishing a diagnosis can be challenging. Some of these conditions are not detected by first‐, second‐ or third‐trimester ultrasound examination but can be diagnosed after birth with physical or cognitive disability and may require surgical/medical intervention or affect quality of life. In addition, clinical features in the prenatal setting do not always correlate with the postnatal presentation. Appendix S1 summarizes the clinical features associated with this set of disorders.

The initial validation of this 30‐gene prenatal screening panel for monogenic disorders evaluated the performance of the assay on 422 maternal plasma samples, 32 of which had genetic variants targeted by the panel^11^. In the current study, we present our clinical experience with NIPT‐SGD in a larger cohort of women referred for testing. We analyze further the screen‐positive rates based on the clinical indications for testing.

## Methods

This study analyzed retrospectively a cohort of pregnancies that were accessioned for NIPT‐SGD between 14 April 2017 to 27 November 2019. The NIPT‐SGD test is available commercially under the brand name Vistara (Natera, Inc., San Carlos, CA, USA). Testing was made available as a clinical service to all types of obstetric providers for singleton pregnancies at ≥ 9 weeks' gestation. Clinicians had access to educational brochures, Natera's provider educational modules, publications and other online resources. Patients were informed by their clinical providers about the benefits and hazards of the NIPT‐SGD test and all patients provided consent for testing. In addition to resources available at some of the practices, pre‐ and post‐test genetic information sessions were available to the patients through Natera's in‐house staff of genetic counselors. Information requested by the laboratory at the time of referral included the presence of abnormal ultrasound findings, family history of SGD, the age of both parents and gestational age. If the pregnant woman was known to be affected by one of the conditions on the panel, that variant was excluded from test interpretation, since the plasma is a mixture of maternal and fetal DNA and differentiating them is challenging. Fetal risk assessment was still available for other variants within that gene and other conditions by which the pregnant woman was not affected. Maternal age ≥ 35 years and paternal age ≥ 40 years were considered to be advanced maternal and paternal age (APA), respectively.

All testing was performed and interpreted in a reference laboratory (Baylor Genetics, Houston, TX, USA) accredited by the College of American Pathologists and Clinical Laboratory Improvement Act. Maternal venous blood (collected in BCT Streck tubes), paternal venous blood (collected in EDTA tubes) or paternal saliva (collected in Oragene tubes) were collected to screen singleton pregnancies ≥ 9 weeks' gestation. Testing was performed on samples that met quality metrics, which included a minimum fetal fraction of 4.5% and minimum next‐generation sequencing (NGS) coverage of at least 97% of the target regions. NIPT‐SGD involved sequencing of coding exons and 10 bp exon/intron boundaries in DNA extracted from maternal plasma, maternal genomic DNA and paternal genomic DNA (trio testing). This process included library construction, target gene enrichment and NGS. A complete description of the assay development and analytical validation has been published previously by Zhang *et al*.^11^. Fetal fraction was determined using a single‐nucleotide polymorphism‐based approach by measuring the paternal allele contribution in the maternal plasma^5^, ^12^. Paternal DNA was also used for quality control and for variant classification purposes.

NGS data from each trio was evaluated in aggregate and variants were curated as pathogenic or likely pathogenic based on the guidelines of the American College of Medical Genetics (ACMG)^13^. Before reporting, positive variants were confirmed by a secondary amplicon‐based NGS assay using deeper sequencing (> 10 000×). Variants of unknown significance were not reported. Confirmatory prenatal or postnatal diagnostic testing was recommended for all screen‐positive patients.

For the purposes of assessing the frequency of SGD with respect to the clinical indications for testing and recognizing that some cases had multiple reasons for referral, we classified each case according to the expected largest risk factor. The order of this classification, from higher to lower risk, was: (1) family history (father affected, carrier or another relative affected by a disorder on the screening panel); (2) presence of an abnormality of the long bones (primarily shortened); (3) cranial/facial abnormality; (4) cardiac defect (all heart defects except echogenic focus); (5) lymphatic system defect (hydrops, pleural effusion, cystic hygroma or increased nuchal translucency); (6) other or unspecified ultrasound abnormality; (7) APA; (8) advanced maternal age (even though this is not an indication that is relevant for NIPT‐SGD, some cases were referred for advanced maternal age); (9) unspecified reason for referral. APA was the assumed indication if the paternal age was ≥ 40 years and no other indication was listed. Similarly, AMA was the assumed indication if the maternal age was ≥ 35 years and no other indication was listed.

Collection of the data in this study was conducted as part of a quality assurance program and the data were deidentified before analysis. This study was determined to be exempt from investigational review board approval (E&I IRB: 20099‐01). In addition to collecting follow‐up data provided voluntarily by some clinics, we actively contacted the clinics of patients with a positive NIPT‐SGD result in order to evaluate the clinical validity of the testing. The dataset presented in this manuscript includes the 32 positive cases reported previously by Zhang *et al*.^11^ and one case published as an individual case report^14^. CIs were derived and statistical comparisons carried out using online calculators (https://measuringu.com/calculators/wald/ and http://vassarstats.net).

## Results

**Figure 1:**
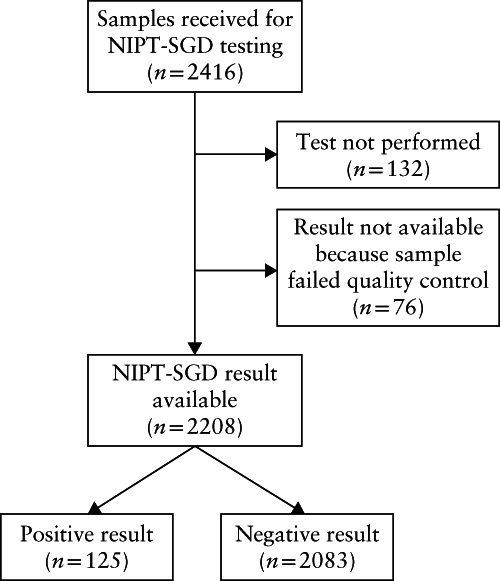
Flowchart of pregnancies referred for non‐invasive prenatal testing for single‐gene disorders (NIPT‐SGD) and outcome of those that received a result and were included in the study.

From 14 April 2017 to 27 November 2019, a total of 2416 cases were accessioned for the NIPT‐SGD 30‐gene panel. Of these, 132 (5.5%) tests were cancelled upon accession due to lack of eligibility (e.g. gestational age too early, twin pregnancy, low sample volume, wrong collection tube or damaged specimen) and a further 76 (3.1%) did not pass the quality control after processing, owing to poor DNA quality, low fetal fraction, low fetal DNA yield after amplification or misidentified parentage. Therefore, results were available for 2208 cases (Figure 1). Table 1 summarizes the indications for NIPT‐SGD and the demographics of the pregnancies with a result.

**Table 1:** Indication for non‐invasive prenatal testing for single‐gene disorders (NIPT‐SGD) and demographics of 2208 pregnancies with a NIPT‐SGD result

Parameter	Value
Indication for testing*	
Family history	132 (6.0)
Abnormal US finding†	514 (23.3)
Advanced paternal age	912 (41.3)
Unspecified/other/advanced maternal age	650 (29.4)
Paternal age (years)	39.5 ± 9.1
Maternal age (years)	34.6 ± 5.9
Gestational age‡	
All indications (weeks)	15.9 ± 6.3
Cases without US abnormality (weeks)	14.1 (9.0–40.1)
Cases with US abnormality (weeks)	22.1 (9.4–38.3)

Data are presented as *n* (%), mean ± SD or mean (range).

* Indications were classified according to a hierarchical prioritization, as described in the Methods.

† Including abnormality of the long bones, cranial/facial abnormality, cardiac defect, lymphatic system defect or other ultrasound (US) abnormality.

‡ Gestational age is given for 2156 cases, as in 52 cases the gestational age provided was incorrect (e.g. blank, 0 or an incorrect number).

Of the 2416 tests accessioned, 56.3% (*n* = 1359) were referred by specialty maternal–fetal medicine (MFM) practices with an available geneticist or genetic counselor, 30.6% (*n* = 739) were referred by general obstetric and gynecological practices (OBGYN) and in 13.2% (*n* = 318) the referral group specialty was unknown. Of the 514 cases with an available result for which the indication for NIPT‐SGD was abnormal ultrasound findings, 74.7% (*n* = 384) were referred by specialty MFM/genetics practices, 13.2% (*n* = 68) by general OBGYN practices and in 12.1% (*n* = 62) the referral group specialty was unknown. Of the 132 cases with an available result for which the indication for NIPT‐SGD was positive family history, 51.5% (*n* = 68) were referred by specialty MFM/genetics practices, 29.5% (*n* = 39) were referred by general OBGYN practices and in 18.9% (*n* = 25) the practice type was unknown.

Of the 2208 cases with a NIPT‐SGD result, 125 (5.7%) tested positive for a SGD. Table 2 summarizes the number of affected pregnancies identified for each of the major groups of conditions included in the NIPT‐SGD panel. Table S1 presents the specific gene variants and additional clinical details for these test‐positive cases. Of the total number of variants identified, 104 were classified as pathogenic and 21 as likely pathogenic. There were 15 variants that were considered novel (i.e. variants for which no published or database information existed to fully assess the phenotype but which, based on the molecular change, could be reasonably inferred as being consequential clinically). These variants were considered likely pathogenic in accordance with the ACMG guidelines^13^ and therefore included in the reported information.

**Table 2:** Disorders identified through non‐invasive prenatal testing for single‐gene disorders (NIPT‐SGD) in 2208 cases with a NIPT‐SGD test result, according to group of condition

Disorder group	Positive cases identified (*n*)	% of all test‐positive cases	% of all results
Skeletal*	73	58.4	3.3
Craniosynostosis†	10	8.0	0.45
Noonan spectrum disorders‡	29	23.2	1.3
Other syndromic disorders§	13	10.4	0.59
Total	125	100	5.7

* Osteogenesis imperfecta (*n* = 35), thanatophoric dysplasia (*n* = 21), achondroplasia (*n* = 14), hypochondroplasia (*n* = 2) and *COL1A2*‐related disorders (*n* = 1).

† General craniosynostosis (*n* = 2) and Apert (*n* = 5), Pfeiffer (*n* = 2) and Crouzon (*n* = 1) syndromes.

‡ Noonan syndrome (*n* = 26), Costello syndrome (*n* = 1), cardiofaciocutaneous syndrome (*n* = 1) and Noonan‐like syndrome with loose anagen hair (*n* = 1).

§ Tuberous sclerosis (*n* = 4), Alagille syndrome (*n* = 2), Cornelia de Lange (*n* = 5), autosomal dominant mental retardation‐5 (*n* = 1) and X‐linked dominant early infantile epileptic encephalopathy‐2 (*n* = 1).

A breakdown of positive NIPT‐SGD results by indication for testing is provided in Table 3. A total of 132 cases were tested because of a family history for one of the conditions on the panel, including pregnancies in which the father was known to be affected, the couple had another affected child or a more distant affected family member existed. The NIPT‐SGD result was positive for 20/132 (15.2%) of these cases and included 17 with an autosomal dominant condition known to be derived from the father, two in which the condition was inherited from the mother (not known at the time of referral) and one with a previous child affected by the same condition. Additionally, in 12 cases, a parent was diagnosed as affected because of a positive NIPT‐SGD result (10 maternal and two paternal discoveries). Clinical confirmation via diagnostic testing was obtained in 50% (5/10) of the maternal cases discovered as a result of NIPT‐SGD testing, and in 2/10 cases the clinic confirmed clinical features suggestive of NSD in both the mother and a previous child. In both paternal cases discovered as a result of NIPT‐SGD, the fetal diagnosis was confirmed by postnatal clinical exam.

**Table 3:** Positive result on non‐invasive prenatal testing for single‐gene disorders (NIPT‐SGD) in 2208 cases, according to indication for referral

Indication for NIPT‐SGD*	Referrals (*n*)	Positive result	
		*n*	% (95% CI)
Positive family history	132	20	15.2 (10.0–22.3)
Abnormal US finding	514	99	19.3 (16.1–22.9)
Long‐bone abnormality	178	60	33.7 (27.2–40.9)
Cranial/facial abnormality	21	6	28.6 (13.6–50.2)
Lymphatic system defect	150	20	13.3 (8.7–19.8)
Cardiac defect	31	4	12.9 (4.5–29.5)
Other or unspecified US finding	134	9	6.7 (3.4–12.4)
Advanced paternal age	912	2	0.2 (0.0–0.9)
Advanced maternal age/unspecified/other	650	4	0.6 (0.2–1.6)
Total	2208	125	5.7 (4.8–6.7)

* Indications were classified according to a hierarchical prioritization, as described in the Methods.

US, ultrasound.

Of the 514 cases that underwent NIPT‐SGD testing because of abnormal ultrasound findings, 99 (19.3%) had a positive result (Table 3). Patients who underwent testing because of an abnormal ultrasound finding were referred significantly later in pregnancy (mean, 22.1 weeks) compared to those who were referred for other reasons (mean, 14.1 weeks) (*P* < 0.001) (Tables 1 and 4). In patients referred for NIPT‐SGD testing due to an abnormal ultrasound finding, the mean gestational age at testing was later for those with a positive compared to those with a negative NIPT‐SGD result (24.3 weeks *vs* 21.5 weeks; *P* < 0.001).

**Table 4:** Timing of referral for non‐invasive prenatal testing for single‐gene disorders (NIPT‐SGD) in 499 pregnancies referred due to abnormal ultrasound findings, overall and according to whether the NIPT‐SGD result was positive or negative

Parameter	Positive result (*n* = 99)	Negative result* (*n* = 400)	All (*n* = 499)
Timing of referral†			
First trimester	7 (7.1)	87 (21.8)	94 (18.8)
Second trimester	62 (62.6)	223 (55.8)	285 (57.1)
Third trimester	30 (30.3)	90 (22.5)	120 (24.0)
Gestational age (weeks)	24.3 (11.4–36.7)	21.5 (9.4–38.3)	22.1 (9.4–38.3)

Data are given as *n* (%) or mean (range).

* Fifteen test‐negative cases referred due to abnormal ultrasound finding had incorrect gestational age and are not reported.

† Trimesters are based on the ACOG guidelines: first trimester, 0–13.9 weeks; second trimester, 14.0–27.9 weeks; third trimester, 28.0–40.9 weeks.

Among the 514 cases that underwent NIPT‐SGD testing because of an abnormal ultrasound finding, the most common primary indication involved shortened or abnormal long bones (178/514; 34.6%). This group also showed the highest rate of positive tests (60/178; 33.7%). Of the 60 positive cases with this reason for referral, 22 were associated with osteogenesis imperfecta, 19 with thanatophoric dysplasia and 13 with achondroplasia/hypochondroplasia. The less common conditions were NSD (*n* = 2), CdLS (*n* = 2), Apert syndrome (*n* = 1) and Alagille syndrome (*n* = 1). A high test‐positive rate (6/21; 28.6%) was also observed for referrals due to cranial/facial defects. Referrals with lymphatic system abnormality (increased nuchal translucency, cystic hygroma, pleural effusion) were also common, comprising 150/514 (29.2%) cases. Of the 20 test‐positive cases in this group, 18 were variants associated with NSD. There was also one case of CdLS and one with osteogenesis imperfecta. Of the 31 (6.0%) referrals with a primary indication of cardiac abnormality, four had positive NIPT‐SGD findings. None of the variants was associated with NSD; the positive results were for tuberous sclerosis (*n* = 3) and CdLS (*n* = 1).

A total of 912 tests were performed for APA (≥ 40 years) and no other specific indication. The APA test‐positive rate was 2/912 (0.2%) (the paternal ages were 55 and 61 years). For comparison, of 650 tests performed in cases with paternal age < 40 years and no other indication for testing, four (0.6%) were positive (paternal ages of 28, 28, 33 and 36 years). Thus, while these data provided no direct evidence for a paternal age effect, APA was the most common feature of the referral population, with an average paternal age of 39.5 years (Table 1). Moreover, among the 99 test‐positive cases that had abnormal ultrasound findings, 18 (18%) also had APA. This would potentially suggest a paternal age effect in this subgroup, but the dataset was too small and heterogeneous for additional investigation into paternal age as a contributing factor.

We attempted to gather information on additional testing and pregnancy outcomes for the 125 NIPT‐SGD positive cases. Among these, seven (5.6%) women had previously undergone amniocentesis or chorionic villus sampling (CVS) with a normal karyotype and/or microarray result. All seven cases had sonographic abnormalities. In addition, 31 (24.8%) cases were known to have received a normal NIPT result. In 30 (24.0%) cases, no additional screening (NIPT, maternal serum screening) or diagnostic testing was pursued prior to the NIPT‐SGD test. For the rest of the positive cases, the information was not available from the clinic, or the case was lost to follow‐up. For NIPT‐SGD negative cases, comprehensive information for related testing was not available. However, at least 840/1661 (50.6%) received NIPT aneuploidy screening (with low‐risk results). This rate of utilization of aneuploidy screening is a minimal estimate because of the incomplete ascertainment.

In 20/125 (16%) cases with a positive NIPT‐SGD result, the specific autosomal dominant disorder was known to be segregated from the father (*n* = 18) or the mother (*n* = 2) and, therefore, confirmatory studies were deemed non‐essential. Of the other 105 test‐positive cases, confirmatory study results were available for 47 (44.8%) cases, of which 34 were confirmed by prenatal or postnatal diagnostic testing, 10 were independently confirmed by postnatal clinical exam and three were confirmed by maternal blood studies. In one additional case, maternal mosaicism was detected by NIPT‐SGD and the risk for each subsequent pregnancy was therefore up to 50%. In this particular case, the maternal variant was not detected by fetal diagnostic testing. The remaining 57 cases either planned to pursue diagnostic testing postnatally or were lost to follow‐up. Among cases with confirmatory follow‐up testing, no false‐positive or false‐negative results were reported.

Among the screen‐positive cases, 18.4% (23/125) reported an adverse outcome, including two cases of intrauterine fetal demise, four stillbirths, 13 neonatal deaths and four preterm deliveries. There were also seven elective pregnancy terminations.

Care providers were queried whether the NIPT‐SGD positive result precipitated pregnancy management changes. This included a change in delivery location (e.g. referral to a high‐risk pregnancy center), change in the delivery plan (e.g. early induction or Cesarean section instead of vaginal delivery), referral to additional specialists or altered post‐delivery plans (including ensuring neonatal intensive care unit availability or opting for palliative comfort care). Of 43 responses received, 17 (39.5%) responded ‘no’ and 26 (60.5%) responded ‘yes’.

## Discussion

In this study, we report on our clinical experience with NIPT‐SGD, which focuses on a specific set of dominantly inherited or *de‐novo* gene variants in 30 genes. In our clinical cohort, enriched for pregnancies at high risk for these disorders, 5.7% (125/2208) tested positive. The highest test‐positive rates were observed for cases tested because of fetal long‐bone abnormalities (33.7%), fetal craniofacial malformations (28.6%), family history of a disorder included in the panel (15.2%), fetal lymphatic abnormalities (13.3%) and fetal cardiac abnormalities (12.9%). In addition to identifying causal gene variants in fetuses with abnormalities, the test detected previously unidentified carrier parents. Among cases with confirmatory follow‐up testing, no false‐positive or false‐negative results were found.

Our results extend those of a validation study by Zhang *et al*.^11^. In that study, a series of 422 cases were evaluated using the same NIPT‐SGD assay and, of the 147 cases with independent confirmatory studies, 20 were true‐positive, 127 were true‐negative and there were no false‐positive or false‐negative results. In another small series, Yan *et al*.^15^ evaluated the same assay in 13 cases with skeletal abnormalities or increased nuchal translucency and identified eight fetuses with monogenic disorders and two affected mothers, with no false‐positive results. With 2208 reported results, this study represents the largest cohort of NIPT‐SGD used in clinical practice.

The genes included in the panel are expected to detect over 80% of all NSD (Appendix S1). Therefore, this testing has an obvious application for pregnancies in which this group of disorders is under consideration due to the presence of polyhydramnios, cystic hygroma, increased nuchal translucency, fetal hydrops or cardiac anomalies^16^. NSD have a combined incidence of 1 in 1000 to 1 in 2500 live births^17^. They represent the second most common genetic cause of congenital heart defect^18^, with affected individuals presenting with multisystemic abnormalities associated with high morbidity and mortality^16^. Prenatal diagnosis is advantageous because altered delivery management and neonatal medical interventions can be beneficial. Among the tests performed due to an indication of ultrasound evidence for lymphatic abnormalities, we found positive results for genes other than those associated typically with NSD. Likewise, within cases with ultrasound evidence for skeletal abnormalities, we found positive results for genes other than those associated typically with osteogenesis imperfecta, thanatophoric dysplasia and achondroplasia (Appendix S1). These results indicate clinical benefit for the use of a broad NIPT‐SGD panel for screening patients suspected of having one of these disorders.

Although the observed test‐positive rate was just 0.4% (6/1562) in the cases without abnormal ultrasound findings or family history (Table 3), NIPT‐SGD could be beneficial for sonographically apparently normal pregnancies. Many of the conditions on the NIPT‐SGD panel are associated with absent or non‐specific prenatal ultrasound findings (Appendix S1). Moreover, NIPT‐SGD can be offered in the first trimester (in conjunction with NIPT to screen for aneuploidy), at which time the associated fetal anatomical abnormalities are typically not visible. In this study, of the 99 sonographically abnormal cases with variants, 30 (30.3%) were identified only after the abnormal ultrasound findings were seen in the third trimester (Table 4), beyond the recommended window for CVS or amniocentesis. A further 62/99 (62.6%) cases were only referred following identification of second‐trimester ultrasound abnormalities, with limited time for a full diagnostic work‐up. NIPT‐SGD could also be more appropriate for pregnancies from older fathers because of the association between *de‐novo* variants and paternal age^19^, although no direct evidence for the association with paternal age could be demonstrated in this study. Friedman^20^ estimated that the risk for *de‐novo* pathogenic single‐gene variants for fathers aged > 40 years was at least 0.3–0.5%. Our dataset was too small and too heterogeneous to allow us to explore the paternal age association in detail.

Limitations of this study need to be emphasized. There were no strict inclusion criteria for access to this testing and, therefore, the referral population was probably enriched for those patients who were perceived by their healthcare providers to be most likely to benefit. Therefore, the cases studied were not necessarily representative of all pregnancies. We do not know how many patients in the study group had undergone additional testing to rule out aneuploidy or copy number variants before NIPT‐SGD, which would affect the proportion of cases with positive results. Similarly, some patients with a family history for one of the disorders included in the panel could have undergone other testing or used NIPT‐SGD to avoid invasive testing. Follow‐up data were not received for all test‐positive cases and post‐delivery studies were not performed in all test‐negative cases; therefore, we cannot exclude the possibility of false‐positive or false‐negative results. Although the performance data from this study indicates that a *de‐novo* or paternally inherited genetic variant can be detected with a high degree of certainty, we urge caution in concluding that the NIPT‐SGD testing is diagnostic. Therefore, we advocate confirmatory testing on samples derived from invasive procedures.

Given that NIPT‐SGD can potentially help detect some affected pregnancies, the question of how NIPT‐SGD could be integrated into prenatal care bears asking. The standard of care currently recommends that pregnant women with abnormal ultrasound findings be offered invasive diagnostic testing through CVS or amniocentesis, with analysis by chromosome microarray^21^. For those cases without an explanatory chromosomal imbalance, panel‐based molecular analysis or exome sequencing (ES) can be considered. NIPT‐SGD cannot be expected to identify the full genome‐wide range of variants potentially identifiable by ES. On the other hand, ES is expensive and often requires 2–4 weeks to obtain results. Our observations with NIPT‐SGD justify additional clinical studies to evaluate the optimal design of the gene panel, define target populations and assess patient acceptability of this testing in various clinical scenarios.

Wider availability of NIPT‐SGD presents additional challenges. Similar to ES, the testing does not identify promoter and deep intronic variants, copy‐number variations and structural rearrangements. Many of the conditions show variable penetrance or expressivity. NIPT‐SGD is currently unsuitable for routine screening for maternally inherited variants. In some cases, the testing will identify an inherited variant not previously known to be present in a parent. Despite these issues, we found that, of cases with a positive NIPT‐SGD result in which feedback was received, 60.5% of clinical providers confirmed that the positive result elicited changes to the pregnancy management. NIPT‐SGD is in its earliest stages of development and considerable potential exists to expand its scope through the sequencing of more genes.

In conclusion, this study demonstrates the potential value of NIPT‐SGD, particularly in cases with abnormal ultrasound findings or with a relevant family history. If implemented correctly with close counseling and monitoring, NIPT‐SGD offers a safe and timely prenatal screening option.

## Supporting information


**Appendix** **S1** Description of disorders included in the non‐invasive prenatal testing for single‐gene disorders (NIPT‐SGD) panel and expected performance of the panel

**Table S1** Gene variants and clinical details for 125 cases with a positive result on non‐invasive prenatal testing for single‐gene disorders (NIPT‐SGD)

## Data Availability

Data is available in the Supplemental files.
