# Supplementary material for: Clinical experience with non‐invasive prenatal screening for single‐gene disorders
Source: Ultrasound Obstet Gynecol. 2022 Jan 5;59(1):33–9. doi: 10.1002/uog.23756 (PMC9302116; doi:10.1002/uog.23756)
Supplement: Supplementary file 1 — Appendix S1 Description of disorders included in the non‐invasive prenatal testing for single‐gene disorders (NIPT‐SGD) panel and expected performance of the panel [file UOG-59-33-s001.docx]

**Appendix** S1 Description of the disorders included in the non-invasive prenatal testing for single-gene disorders (NIPT-SGD) panel and expected performance of the panel

The following is a brief description of the disorders included in the NIPT-SGD panel. The Tables summarize the genes tested, the proportion of cases that are expected to be attributable to variants in the gene (column a); the expected performance of the test as described by Zhang et al., (column b)^1^; and therefore, the approximate expected net detection rate (column c, based on the product of the data in columns a and b).

**Skeletal Disorders**

Skeletal disorders included in the NIPT-SGD panel include conditions that can range from mild to lethal. For example, Osteogenesis Imperfecta (OI), caused by variants in the *COL1A1* and *COL1A2* gene, has a spectrum of presentation, from a few fractures in childhood to death in the prenatal/neonatal period.^2^ Likewise, disorders involving the *FGFR3* gene can lead to a range of disease severity, from classic achondroplasia to lethal thanatophoric dysplasia, depending on the specific variant present.^3-5^ There is a high rate of de novo variants arising in the *FGFR3* gene and the rate is increased for older males.

**Table 1. Skeletal Disorders**

| **Condition** | **Genes** | **% of condition caused by gene (a)** | **Expected test performance (b)** | **Net detection rate (c)** |
| --- | --- | --- | --- | --- |
| Osteogenesis Imperfecta | *COL1A1 and COL1A2* | 90%^6 7^ | >92% | **82.8** |
| Ehlers-Danlos syndrome | *COL1A1 and COL1A2* | unknown | >92% | **unknown** |
| Hypochondroplasia | *FGFR3* | 70-90%^8^ | >96% | **67.2** |
| Achondroplasia | *FGFR3* | 99%^5^ | >96% | **95.0** |
| Thanatophoric dysplasia | *FGFR3* | 90-99%^4^ | >96% | **86.4** |
| CATSHL | *FGFR3* | 100%^9^ | >96% | **96** |
| Crouzon syndrome with acanthosis nigricans | *FGFR3* | 100%^10^ | >96% | **96** |
| Muenke syndrome | *FGFR3* | 100%^11^ | >96% | **96** |

**Craniosynostosis syndromes**

Craniosynostosis disorders have significant overlap in their clinical features, and include Crouzon syndrome, Apert syndrome, and Pfeiffer syndrome.^12-14^ Craniosynostosis can require surgical intervention in many of the affected individuals, while some affected individuals will have symptoms in multiple systems that could need additional medical support. Although a degree of variability exists, individuals with certain types of craniosynostosis may have intellectual or learning disabilities. Craniosynostosis is usually not detectable through prenatal ultrasound until at least the third trimester although syndromic cases may be identified somewhat earlier.^14^ Some cases are attributable to variants in *FGFR3* and *TWIST* genes.^15^

**Table 2. Craniosynostosis syndromes**

| **Condition** | **Genes** | **% of condition caused by gene (a)** | **Expected test performance (b)** | **Net detection rate (c)** |
| --- | --- | --- | --- | --- |
| Pfeiffer syndrome | *FGFR2* | >95%^16^ | >96% | **91.2** |
| Crouzon syndrome | *FGFR2* | 100%^16^ | >96% | **96.0** |
| Apert syndrome | *FGFR2* | >98%^17^ | >96% | **94.1** |
| Antley-Bixler syndrome without genital anomalies or disordered steroidogenesis | *FGFR2* | 100%^18^ | >96% | **96** |
| Jackson-Weiss syndrome | *FGFR2* | 100%^16^ | >96% | **96** |

**Noonan Spectrum Disorders**

Noonan spectrum disorders (NSDs), sometimes called “rasopathies”, are a heterogenous family of mostly autosomal dominant disorders involving genes in the RAS/MAPK cell signaling pathway.^19-21^ Clinical features in individuals with NSDs vary widely, and include birth defects, characteristic dysmorphic features, developmental delay, bleeding disorders, and predisposition to malignancies. NSDs are frequently considered as part of the differential diagnosis when prenatal ultrasound shows increased nuchal translucency (NT), cystic hygroma, hydrops or edema, congenital heart disease (CHD), pleural effusion, ascites, polyhydramnios, relative macrocephaly, renal and skeletal anomalies.

**Table 3. Noonan Spectrum Disorders**

| **Condition** | **Genes** | **% of condition caused by gene (a)** | **Expected test performance (b)** | **Net detection rate (c)** |
| --- | --- | --- | --- | --- |
| Noonan syndrome-1/  LEOPARD syndrome  /cancers | *PTPN11* | 50%^19^ | >96% | **48** |
| Noonan syndrome-4 | *SOS1* | 10-13%^19^ | >96% | **9.6** |
| Noonan syndrome-5/  LEOPARD syndrome 2 | *RAF1* | 5%^19^ | >96% | **4.8** |
| Noonan syndrome-8 | *RIT1* | 5%^19^ | >96% | **4.8** |
| Noonan syndrome/ cancers/ Cardiofaciocutaneous syndrome | *KRAS* | <5%^19^ | >96% | **4.8** |
| Cardiofaciocutaneous syndrome 1 | *BRAF* | <2%^19^ | >96% | **1.92** |
| Cardiofaciocutaneous syndrome 3 | *MAP2K1* | <2%^19^ | >96% | **1.92** |
| Cardiofaciocutaneous syndrome 4 | *MAP2K2* | unknown | >96% | **unknown** |
| Costello syndrome,  Noonan syndrome | *HRAS* | <1%^19^ | >92% | **0.92** |
| Noonan syndrome-like disorder w or w/o juvenile myelomonocytic leukemia (NSLL) | *CBL* | <1%^19^ | >94% | **0.94** |
| Noonan syndrome-6/  cancers | *NRAS* | <1%^19^ | >96% | **0.96** |
| Noonan syndrome-like disorder with loose anagen hair | *SHOC2* | <1%^19^ | >96% | **0.96** |
| Noonan syndrome-9 | *SOS2* | <1%^19^ | >96% | **0.96** |
| **ALL NOONAN COMBINED** |  |  |  | **81.5** |

**Other syndromic disorders**

Cornelia de Lange syndrome (CdLS) is characterized by multiple congenital anomalies, including limb deficiencies, distinctive facial features, hypertrichosis, prenatal and postnatal growth deficiency, developmental delay, and behavioral issues. Some individuals can have a mild presentation and others are more severe.^22^ Variants in several genes that affect chromatin structure are causal.^23^

Alagille syndrome is a widely variable disorder. The most apparent clinical feature of this condition is fewer small bile ducts in the liver, which results in a buildup of bile in the liver. Additional features can include skeletal findings, ophthalmological abnormalities, distinct facies, heart defects, kidney and vascular abnormalities.^24^ Genetic variants causing loss of function in the *JAG1,* or *NOTCH2* genes cause this disorder.

Tuberous Sclerosis is caused by variants in the *TSC1* and *TSC2* genes. The disorder is characterized by multiple benign tumors throughout different parts of the body including the skin, brain, kidneys, heart and lungs.^25, 26^

Epileptic encephalopathy, early infantile 2, is a developmental disorder with common features including infantile onset of seizures and severe developmental delay.^27^ Additional findings can include gastrointestinal and sleep issues. The disorder shows an X-linked dominant pattern of inheritance with mutation in the *CDKL5* gene. Additional findings can include gastrointestinal and sleep issues. This disorder is not expected to be identified through prenatal ultrasound.

SYNGAP related Intellectual Disability includes moderate to severe developmental delays and intellectual disability, seizures, autism spectrum disorder, and characteristic behavioral abnormalities.^28^ This disorder is not expected to be identified through prenatal ultrasound. The disorder is associated with the Synaptic Ras GTPase-activating protein 1, *SYNGAP1*, also known as *Ras-GAP1*.

Sotos syndrome is characterized by prenatal and postnatal bone overgrowth, behavioral/intellectual disabilities, and characteristic craniofacial abnormalities. Other features can include heart and kidney abnormalities, seizures, hyper mobile joints, and scoliosis.^29^ Some cases come to attention through prenatal ultrasound through suspected overgrowth. Most cases are attributable to de novo variants in the *NSD1* gene.

CHARGE syndrome can affect many different parts of the body. Cardinal features include coloboma, heart defects, choanal atresia, fetal growth retardation, as well as development, genital and ear abnormalities. In addition, there are several features of CHARGE syndrome that have been linked to alterations in the CHD7 gene.^30^ These features include abnormalities of the brain, kidneys, limbs, scoliosis, and immune system defects. Although individual features of this disorder may be identified through prenatal ultrasound, a prenatal diagnosis is not generally made (except when exome sequencing is offered).

Rett syndrome is a neurodevelopmental X-linked disorder that affects predominantly females. Features of Rett syndrome can vary and include hypotonia and acquired microcephaly with decline of developmental progress over time, stereotypic hand movements, behavior, balance and breathing problems as well as intellectual disability. Classic Rett syndrome is attributable to variants in the MECP2 gene. ^37, 38^

**Table 4. Other syndromic disorders**

| **Condition** | **Genes** | **% of condition caused by gene (a)** | **Expected test performance (b)** | **Net detection rate (c)** |
| --- | --- | --- | --- | --- |
| Cornelia de Lange syndrome 1 | *NIPBL* | 80%^31^ | >94% | **75.2** |
| Cornelia de Lange syndrome 2 | *SMC1A* | 5%^31^ | >96% | **4.8** |
| Cornelia de Lange syndrome 3 | *SMC3* | 1-2%^31^ | >96% | **0.69** |
| Cornelia de Lange syndrome 4 | *RAD21* | <1%^31^ | >53% | **0.53** |
| Cornelia de Lange syndrome 5 | *HDAC8* | 4%^13^ | >66% | **2.64** |
| CHARGE syndrome | *CHD7* | 90%^32^ | >91% | **81.9** |
| Alagille syndrome | *JAG1* | 94.3%^33^ | >86% | **80.84** |
| Tuberous Sclerosis | *TSC2* | 69%^34^ | >91% | **62.79** |
| Tuberous Sclerosis | *TSC1* | 26%^34^ | >92% | **23.92** |
| Epileptic encephalopathy, early infantile 2 | *CDKL5* | unknown | >84% | **unknown** |
| SYNGAP Intellectual disability | *SYNGAP1* | 89%^28^ | >86% | **76.54** |
| Sotos syndrome | *NSD1* | 90%^35^ | >47% | **42.3** |
| Rett syndrome | *MECP2* | 90-95%^36^ | >78% | **70.2** |

**Supplemental References**

1. Zhang J, Li J, Saucier JB, Feng Y, Jiang Y, Sinson J, McCombs AK, Schmitt ES, Peacock S, Chen S, Dai H, Ge X, Wang G, Shaw CA, Mei H, Breman A, Xia F, Yang Y, Purgason A, Pourpak A, Chen Z, Wang X, Wang Y, Kulkarni S, Choy KW, Wapner RJ, Van den Veyver IB, Beaudet A, Parmar S, Wong LJ, Eng CM. Non-invasive prenatal sequencing for multiple Mendelian monogenic disorders using circulating cell-free fetal DNA. *Nat Med* 2019; **25**: 439-447.

2. Chetty M, Roomaney IA, Beighton P. The evolution of the nosology of osteogenesis imperfecta. *Clin Genet* 2021; **99**: 42-52.

3. Bober MB, Bellus GA, Nikkel SM, Tiller GE. Hypochondroplasia. 1999 [updated 2020]. In *GeneReviews((R))*. Adam MP, Ardinger HH, Pagon RA, Wallace SE, Bean LJH, Mirzaa G, Amemiya A (eds). Seattle (WA), 1993.

4. French T, Savarirayan R. Thanatophoric Dysplasia. 2004 [updated 2020]. In *GeneReviews((R))*. Adam MP, Ardinger HH, Pagon RA, Wallace SE, Bean LJH, Mirzaa G, Amemiya A (eds). Seattle (WA), 1993.

5. Legare JM. Achondroplasia. 1998 [updated 2020]. In *GeneReviews((R))*. Adam MP, Ardinger HH, Pagon RA, Wallace SE, Bean LJH, Mirzaa G, Amemiya A (eds). Seattle (WA), 1993.

6. Valadares ER, Carneiro TB, Santos PM, Oliveira AC, Zabel B. What is new in genetics and osteogenesis imperfecta classification? *J Pediatr (Rio J)* 2014; **90**: 536-541.

7. Colombi M, Dordoni C, Venturini M, Zanca A, Calzavara-Pinton P, Ritelli M. Delineation of Ehlers-Danlos syndrome phenotype due to the c.934C>T, p.(Arg312Cys) mutation in COL1A1: Report on a three-generation family without cardiovascular events, and literature review. *A J Med Genet Part A* 2017; **173**: 524-530.

8. Bober MB, Bellus GA, Nikkel SM, Tiller GE. Hypochondroplasia. 1999 [updated 2020]. In *GeneReviews((R))*. Adam MP, Ardinger HH, Pagon RA, Wallace SE, Bean LJH, Mirzaa G, Amemiya A (eds). Seattle (WA), 1993.

9. Online Mendelian Inheritance in Man (OMIN). Camptodactyly, tall stature, and hearing loss syndrome; CATSHLS. https://omim.org/entry/610474 Accessed 03/29/2021.

10. Online Mendelian Inheritance in Man (OMIN). Crouzon syndrome with acanthosis nigricans; CAN. https://www.omim.org/entry/612247 Accessed 03/29/2021.

11. Kruszka P, Addissie YA, Agochukwu NB, Doherty ES, Muenke M. Muenke Syndrome. 2006 [updated 2016]. In *GeneReviews((R))*. Adam MP, Ardinger HH, Pagon RA, Wallace SE, Bean LJH, Mirzaa G, Amemiya A (eds). Seattle (WA), 1993.

12. Conrady CD, Patel BC. Crouzon Syndrome. In *StatPearls*. Treasure Island (FL), 2021.

7. Conrady CD, Patel BC, Sharma S. Apert Syndrome. In *StatPearls*. Treasure Island (FL), 2021.

13. J MD, Winters R. Pfeiffer Syndrome. In *StatPearls*. Treasure Island (FL), 2021.

14. Giancotti A, D'Ambrosio V, Marchionni E, Squarcella A, Aliberti C, La Torre R, Manganaro L, Pizzuti A, PECRAM Study Group. Pfeiffer syndrome: literature review of prenatal sonographic findings and genetic diagnosis. *J Mat Fetal Neonat Med.* 2017; **30**: 2225-2231.

15. Armand T, Schaefer E, Di Rocco F, Edery P, Collet C, Rossi M. Genetic bases of craniosynostoses: An update. *Neurochirurgie* 2019; **65**: 196-201.

16. Wenger T, Miller D, Evans K. FGFR Craniosynostosis Syndromes Overview. 1998 [updated 2020]. In *GeneReviews((R))*. Adam MP, Ardinger HH, Pagon RA, Wallace SE, Bean LJH, Mirzaa G, Amemiya A (eds). Seattle (WA), 1993.

17. Bochukova EG, Roscioli T, Hedges DJ, Taylor IB, Johnson D, David DJ, Deininger PL, Wilkie AO. Rare mutations of FGFR2 causing Apert syndrome: identification of the first partial gene deletion, and an Alu element insertion from a new subfamily. *Hum Mutat* 2009; **30**: 204-211.

18. Online Mendelian Inheritance in Man OMIN. Antley-Bixler syndrome without genital anomalies or disordered steroidogenesis; ABS2. https://www.omim.org/entry/207410 Accessed 03/29/2021.

19. Allanson JE, Roberts AE. Noonan Syndrome. In *GeneReviews((R))*. 2001 [updated 2019]. Adam MP, Ardinger HH, Pagon RA, Wallace SE, Bean LJH, Mirzaa G, Amemiya A (eds). Seattle (WA), 1993.

20. Noonan JA. Noonan syndrome. An update and review for the primary pediatrician. *Clin Pediat* 1994; **33**: 548-555.

21. Marino B, Digilio MC, Toscano A, Giannotti A, Dallapiccola B. Congenital heart diseases in children with Noonan syndrome: An expanded cardiac spectrum with high prevalence of atrioventricular canal. *J Pediat* 1999; **135**: 703-706.

22. Cascella M, Muzio MR. Cornelia de Lange Syndrome. In *StatPearls*. Treasure Island (FL), 2021.

23. Avagliano L, Parenti I, Grazioli P, Di Fede E, Parodi C, Mariani M, Kaiser FJ, Selicorni A, Gervasini C, Massa V. Chromatinopathies: A focus on Cornelia de Lange syndrome. *Clin Genet* 2020; **97**: 3-11.

24. Ayoub MD, Kamath BM. Alagille Syndrome: Diagnostic Challenges and Advances in Management. *Diagn (Basel)* 2020; **10:** 11.

25. Mallela K, Kumar A. Role of TSC1 in physiology and diseases. *Mol Cell Biochem* 2021. DOI: 10.1007/s11010-021-04088-3.

26. Zamora EA, Aeddula NR. Tuberous Sclerosis. In *StatPearls*. Treasure Island (FL), 2021.

27. Olson HE, Demarest ST, Pestana-Knight EM, Swanson LC, Iqbal S, Lal D, Leonard H, Cross JH, Devinsky O, Benke TA. Cyclin-Dependent Kinase-Like 5 Deficiency Disorder: Clinical Review. *Pediatr Neurol* 2019; **97**: 18-25.

28. Holder JL, Jr., Hamdan FF, Michaud JL. SYNGAP1-Related Intellectual Disability. In *GeneReviews((R))*.Adam MP, Ardinger HH, Pagon RA, Wallace SE, Bean LJH, Mirzaa G, Amemiya A (eds). Seattle (WA), 1993.

29. Brioude F, Toutain A, Giabicani E, Cottereau E, Cormier-Daire V, Netchine I. Overgrowth syndromes - clinical and molecular aspects and tumour risk. *Nat Rev Endocrinol* 2019; **15**: 299-311.

30. Meisner JK, Martin DM. Congenital heart defects in CHARGE: The molecular role of CHD7 and effects on cardiac phenotype and clinical outcomes. *A J Med Genet Part C, Semi Med Genet* 2020; **184**: 81-89.

31. Deardorff MA, Noon SE, Krantz ID. Cornelia de Lange Syndrome. 2005 [updated 2020]. In *GeneReviews((R))*. Adam MP, Ardinger HH, Pagon RA, Wallace SE, Bean LJH, Mirzaa G, Amemiya A (eds). Seattle (WA), 1993.

32. National Organization for Rare Disorders (NORD). CHARGE syndrome. https://rarediseases.org/rare-diseases/charge-syndrome/ Accessed 03/29/2021.

33. Spinner NB, Gilbert MA, Loomes KM, Krantz ID. Alagille Syndrome. 2000 [updated 2019]. In *GeneReviews((R))*. Adam MP, Ardinger HH, Pagon RA, Wallace SE, Bean LJH, Mirzaa G, Amemiya A (eds). Seattle (WA), 1993.

34. Northrup H, Koenig MK, Pearson DA, Au KS. Tuberous Sclerosis Complex. 1999 [updated 2020]. In *GeneReviews((R))*. Adam MP, Ardinger HH, Pagon RA, Wallace SE, Bean LJH, Mirzaa G, Amemiya A (eds). Seattle (WA), 1993.

35. National Organization for Rare Disorders (NORD). Sotos syndrome. https://rarediseases.org/rare-diseases/sotos-syndrome/ Accessed 03/29/2021.

36. Weaving LS, Ellaway CJ, Gecz J, Christodoulou J. Rett syndrome: clinical review and genetic update. *J Med Genet* 2005; **42**: 1-7.

37. National Organization for Rare Disorders. Rett Syndrome; https://rarediseases.org/rare-diseases/rett-syndrome. Accessed 05/13/2021.

38. MedlinePlus. Rett Syndrome; https://medlineplus.gov/genetics/condition/rett-syndrome. Accessed 05/13/2021.
