# Supplementary material for: Clinical experience with non‐invasive prenatal screening for single‐gene disorders
Source: Ultrasound Obstet Gynecol. 2022 Jan 5;59(1):33–9. doi: 10.1002/uog.23756 (PMC9302116; doi:10.1002/uog.23756)
Supplement: Supplementary file 2 — Table S1 Gene variants and clinical details for 125 cases with a positive result on non‐invasive prenatal testing for single‐gene disorders (NIPT‐SGD) [file UOG-59-33-s002.docx]

**Table S1** Gene variants and clinical details for 125 cases with a positive result on non-invasive prenatal testing for single-gene disorders (NIPT-SGD)

| **Case** | | **Indication** | **Ultrasound category  (if applicable)** | **Gestational age**  **(weeks)** | **Maternal age**  **(years)** | **Paternal age**  **(years)** | **Gene** | **Variant** | **Curation (P/LP)*** | **Condition** | **Inheritance  (if applicable, maternal or paternal)** | **Type of confirmatory testing** | **Pregnancy outcome** |
| --- | --- | --- | --- | --- | --- | --- | --- | --- | --- | --- | --- | --- | --- |
| 1 | Positive Family History | | - | 10.4 | 26 | 29 | *PTPN11* | c.836A>G (p.Y279C) | P | Noonan spectrum disorder | Paternal | Known affected parent | Full term delivery |
| 2 | Positive Family History | | - | 13.3 | 35 | 40 | *JAG1* | c.1446_1448delinsC (p.H483Lfs*2) | P | Alagille syndrome | Paternal | Known affected parent | - |
| 3 | Positive Family History | | - | 13 | 32 | 32 | *FGFR2* | c. 1032G>A(p.A344A) | P | Crouzon syndrome | Paternal | Known affected parent | - |
| 4 | Positive Family History | | - | 13.3 | 19 | 21 | *COL1A1* | c.3225delC (p.G1076Vfs*32) | P | Osteogenesis imperfecta | Paternal | Parental blood, Postnatal clinical exam | Full term delivery |
| 5 | Positive Family History | | - | 12.6 | 23 | 29 | *COL1A2* | c.1513G>A (p.G505S) | LP | Osteogenesis imperfecta | Paternal | Known affected parent | Full term delivery |
| 6 | Positive Family History | | - | 9.5 | 26 | 29 | *PTPN11* | c.922A>G (p.N308D) | P | Noonan spectrum disorder | Paternal | Postnatal blood | Full term delivery |
| 7 | Positive Family History | | - | 13 | 40 | 42 | *PTPN11* | c.329A>C (p.110E>A) | P | Noonan spectrum disorder | Paternal | Known affected parent | Full term delivery |
| 8 | Positive Family History | | - | 15.6 | 24 | 26 | *PTPN11* | c.188A>G (p.Y63C) | P | Noonan spectrum disorder | Paternal | Parental blood | - |
| 9 | Positive Family History | | - | 13.4 | 20 | 24 | *COL1A1* | c.3235G>A (p.G1079S) | P | Osteogenesis imperfecta | Paternal | Postnatal clinical exam | Full term delivery |
| 10 | Positive Family History | | - | 11.3 | 28 | 34 | *PTPN11* | c.922A>G (p.N308D) | P | Noonan spectrum disorder | Paternal | Parental blood | - |
| 11 | Positive Family History | | - | 10.4 | 32 | 32 | *FGFR3* | c.1138G>A (p.G380R) | P | Achondroplasia | Paternal | Chorionic villus sampling | Full term delivery |
| 12 | Positive Family History | | - | 12.4 | 30 | 31 | *COL1A1* | c.3076C>T (p.R1026*) | P | Osteogenesis imperfecta | Paternal | Postnatal blood | Preterm delivery |
| 13 | Positive Family History | | - | 14.1 | 29 | 35 | *COL1A1* | c.976G>C (p.G326R) | P | Osteogenesis imperfecta | Paternal | Known affected parent | - |
| 14 | Positive Family History | | - | 13 | 40 | 40 | *PTPN11* | c.209A>G (p.K70R) | LP | Noonan spectrum disorder | Paternal | Known affected parent | Full term delivery |
| 15 | Positive Family History | | - | 16 | 36 | 42 | *COL1A1* | c.804+1G>A | P | Osteogenesis imperfecta | Maternal | Parental blood | Full term delivery |
| 16 | Positive Family History | | - | 12.6 | 20 | 21 | *COL1A1* | c.386delC (p.P129Lfs*136) | P | Osteogenesis imperfecta | Maternal | Known affected parent | - |
| 17 | Positive Family History | | - | 19 | 31 | 33 | *FGFR3* | c.1949A>C(p.K650T) | P | Hypochondroplasia | Paternal | Postnatal blood | Full term delivery |
| 18 | Positive Family History, Abnormal Ultrasound | | Skeletal | 28.1 | 32 | 46 | *COL1A1* | c.1081C>T (p.R361*) | P | Osteogenesis imperfecta | Paternal | Parental blood | - |
| 19 | Positive Family History, Abnormal Ultrasound | | Skeletal | 32.9 | 29 | 32 | *COL1A1* | c.578dupC (p.G194Wfs*14) | P | Osteogenesis imperfecta | Paternal | Known affected parent | Preterm delivery |
| 20 | Positive Family History, Abnormal Ultrasound | | Cardiac | 25.6 | 24 | 25 | *TSC1* | c.1960C>T (p.Q654*) | P | Tuberous sclerosis | Paternal | Known affected parent | Stillbirth |
| 21 | Abnormal Ultrasound | | Skeletal | 34.7 | 33 | 38 | *COL1A1* | c.887G>C (p.G296A) | LP | Osteogenesis imperfecta | Paternal | Postnatal clinical exam | - |
| 22 | Abnormal Ultrasound | | Skeletal | 29 | 28 | 38 | *JAG1* | c.2429C>T p.P810L | LP | Alagille syndrome | Maternal | Parental blood | - |
| 23 | Abnormal Ultrasound | | Skeletal | 20.7 | 35 | 38 | *COL1A2* | c..2936G>T (p.G979V) | LP | Osteogenesis imperfecta | Maternal | Parental blood | - |
| 24 | Abnormal Ultrasound | | Skeletal | 20.3 | 23 | 20 | *COL1A2* | c.3267+1G>C | LP | Osteogenesis imperfecta | Maternal | - | - |
| 25 | Abnormal Ultrasound | | Skeletal | 26.1 | 20 | 33 | *COL1A2* | c.2684G>A (p.G895D) | P | Osteogenesis imperfecta | - | Postnatal clinical exam | Postnatal demise |
| 26 | Abnormal Ultrasound | | Skeletal | 30.3 | 22 | 34 | *COL1A2* | c.2503G>A (p.G835S) | P | Osteogenesis imperfecta | - | - | - |
| 27 | Abnormal Ultrasound | | Skeletal | 16 | 34 | 35 | *FGFR3* | c.742C>T (p.R248C) | P | Thanatophoric dysplasia | - | - | Elective termination |
| 28 | Abnormal Ultrasound | | Skeletal | 19.3 | 28 | 28 | *FGFR3* | c.1948A>G (p.K650E) | P | Thanatophoric dysplasia | - | - | Postnatal demise |
| 29 | Abnormal Ultrasound | | Skeletal | 21.1 | 27 | 32 | *FGFR3* | c.2419T>G (p.*807G) | P | Thanatophoric dysplasia | - | - | - |
| 30 | Abnormal Ultrasound | | Skeletal | 19.9 | 43 | 44 | *FGFR3* | c.742C>T (p.R248C) | P | Thanatophoric dysplasia | - | - | Postnatal demise |
| 31 | Abnormal Ultrasound | | Skeletal | 27.3 | 33 | 35 | *COL1A1* | c.1678G>A (p.G560S) | P | Osteogenesis imperfecta | - | Amniocentesis | - |
| 32 | Abnormal Ultrasound | | Skeletal | 22.4 | 33 | 38 | *FGFR3* | c.742C>T (p.R248C) | P | Thanatophoric dysplasia | - | - | Postnatal demise |
| 33 | Abnormal Ultrasound | | Skeletal | 20.4 | 29 | 30 | *COL1A1* | c.3425G>T (p.G1142V) | LP | Osteogenesis imperfecta | - | Products of conception | In utero fetal demise |
| 34 | Abnormal Ultrasound | | Skeletal | 29.7 | 41 | 42 | *COL1A2* | c.3034G>A (p.G1012S) | P | Osteogenesis imperfecta | - | Postnatal blood | - |
| 35 | Abnormal Ultrasound | | Skeletal | 29 | 33 | 33 | *FGFR3* | c.1138G>A (p.G380R) | P | Achondroplasia | - | Postnatal clinical exam | Full term delivery |
| 36 | Abnormal Ultrasound | | Skeletal | 17 | 28 | 29 | *COL1A1* | c.4339delG (p.V1447Lfs*79) | LP | Osteogenesis imperfecta | - | - | - |
| 37 | Abnormal Ultrasound | | Skeletal | 24.7 | 32 | 35 | *FGFR3* | c.1111A>T (p.S371C) | P | Thanatophoric dysplasia | - | - | Postnatal demise, Preterm delivery |
| 38 | Abnormal Ultrasound | | Skeletal | 26.2 | 36 | 41 | *NIPBL* | c.7439_7440delGA (p.R2480Kfs*5) | P | Cornelia de Lange | - | Postnatal blood | Full term delivery |
| 39 | Abnormal Ultrasound | | Skeletal | 20.2 | 22 | 22 | *FGFR3* | c.1118A>G (p.Y373C) | P | Thanatophoric dysplasia | - | - | In utero fetal demise |
| 40 | Abnormal Ultrasound | | Skeletal | 20 | 33 | 34 | *COL1A2* | c.3034G>A (p.G1012S) | P | Osteogenesis imperfecta | - | - | - |
| 41 | Abnormal Ultrasound | | Skeletal | 29.9 | 35 | 40 | *FGFR3* | c.1138G>A (p.G380R) | P | Achondroplasia | - | - | Full term delivery |
| 42 | Abnormal Ultrasound | | Skeletal | 20 | 37 | 40 | *FGFR3* | c.742C>T (p.R248C) | P | Thanatophoric dysplasia | - | Products of conception | Elective termination |
| 43 | Abnormal Ultrasound | | Skeletal | 19.7 | 42 | 41 | *FGFR3* | c.1108G>T (p.G370C) | P | Thanatophoric dysplasia | - | Postnatal blood | Full term delivery |
| 44 | Abnormal Ultrasound | | Skeletal | 18.7 | 29 | 29 | *COL1A1* | c.2083G>T (p.G695C) | LP | Osteogenesis imperfecta | - | Amniocentesis | - |
| 45 | Abnormal Ultrasound | | Skeletal | 20.3 | 26 | 31 | *COL1A2* | c.2657G>T (p.G886V) | LP | Osteogenesis imperfecta | - | Amniocentesis | Stillbirth |
| 46 | Abnormal Ultrasound | | Skeletal | 32.2 | 25 | 25 | *FGFR3* | c.1138G>A (p.G380R) | P | Achondroplasia | - | - | - |
| 47 | Abnormal Ultrasound | | Skeletal | 21.1 | 27 | 33 | *FGFR3* | c.2420G>C (p.*807S) | P | Thanatophoric dysplasia | - | - | - |
| 48 | Abnormal Ultrasound | | Skeletal | 17.9 | 30 | 31 | *PTPN11* | c.172A>T (p.N58Y) | LP | Noonan spectrum disorder | - | - | - |
| 49 | Abnormal Ultrasound | | Skeletal | 29.4 | 32 | 36 | *COL1A2* | c.2260G>T (p.G754C) | P | Osteogenesis imperfecta | - | Postnatal clinical exam | Full term delivery |
| 50 | Abnormal Ultrasound | | Skeletal | 36.1 | 25 | 25 | *COL1A1* | c.1444G>A (p.G482R) | P | Osteogenesis imperfecta | - | Postnatal blood | Postnatal demise |
| 51 | Abnormal Ultrasound | | Skeletal | 34.7 | 29 | 29 | *FGFR3* | c.1138G>A(p.G380R | P | Achondroplasia | - | Postnatal blood | Full term delivery |
| 52 | Abnormal Ultrasound | | Skeletal | 25.6 | 34 | 35 | *FGFR3* | c.2416_*3delACGTGAAGG (p.T806delins100) | P | Thanatophoric dysplasia | - | - | - |
| 53 | Abnormal Ultrasound | | Skeletal | 19.3 | 19 | 19 | *COL1A1* | c.2155G>A(p.G719S) | P | Osteogenesis imperfecta | - | Postnatal blood | Full term delivery |
| 54 | Abnormal Ultrasound | | Skeletal | 28.3 | 34 | 36 | *FGFR3* | c.1138G>A (p.G380R) | P | Achondroplasia | - | Postnatal blood | Full term delivery |
| 55 | Abnormal Ultrasound | | Skeletal | 24.6 | 19 | 21 | *FGFR3* | c.2421A>G (p.*807W) | P | Thanatophoric dysplasia | - | - | - |
| 56 | Abnormal Ultrasound | | Skeletal | 27.3 | 32 | 33 | *FGFR3* | c.1138G>A (p.G380R) | P | Achondroplasia | - | - | - |
| 57 | Abnormal Ultrasound | | Skeletal | 16.3 | 37 | 36 | *FGFR3* | c.742C>T (p.R248C) | P | Thanatophoric dysplasia | - | - | Postnatal demise, Preterm delivery |
| 58 | Abnormal Ultrasound | | Skeletal | 34.3 | 34 | 32 | *FGFR3* | c.1138G>A (p.G380R) | P | Achondroplasia | - | - | - |
| 59 | Abnormal Ultrasound | | Skeletal | 22.1 | 30 | 46 | *FGFR3* | c.742C>T (p.R248C) | P | Thanatophoric dysplasia | - | - | - |
| 60 | Abnormal Ultrasound | | Skeletal | 32.9 | 17 | 20 | *COL1A1* | c.1715G>C (p.G572A) | LP | Osteogenesis imperfecta | - | - | Full term delivery |
| 61 | Abnormal Ultrasound | | Skeletal | 20.7 | 25 | 29 | *COL1A1* | c.2784delT (p.G929Afs*179) | P | Osteogenesis imperfecta | - | - | - |
| 62 | Abnormal Ultrasound | | Skeletal | 22 | 29 | 35 | *FGFR3* | c.742C>T (p.R248C) | P | Thanatophoric dysplasia | - | - | - |
| 63 | Abnormal Ultrasound | | Skeletal | 21.4 | 21 | 39 | *FGFR3* | c.742C>T(p.R248C) | P | Thanatophoric dysplasia | - | - | Postnatal demise |
| 64 | Abnormal Ultrasound | | Skeletal | 25.9 | 26 | 28 | *COL1A2* | c.856G>A (p.G286S) | P | Osteogenesis imperfecta | - | - | Full term delivery |
| 65 | Abnormal Ultrasound | | Skeletal | 29.9 | 38 | 46 | *HRAS* | c.34G>A (p.G12S) | P | Noonan spectrum disorder | - | Postnatal blood | Full term delivery |
| 66 | Abnormal Ultrasound | | Skeletal | 24 | 35 | 41 | *COL1A1* | c.1823G>A(p.G608D) | LP | Osteogenesis imperfecta | - | - | Stillbirth |
| 67 | Abnormal Ultrasound | | Skeletal | 23.9 | 23 | 24 | *COL1A2* | c.982G>A (p.G328S) | P | Osteogenesis imperfecta | - | - | - |
| 68 | Abnormal Ultrasound | | Skeletal | 18.6 | 33 | 33 | *FGFR3* | c.742C>T (p.R248C) | P | Thanatophoric dysplasia | - | - | Elective termination |
| 69 | Abnormal Ultrasound | | Skeletal | 33.9 | 37 | 39 | *FGFR3* | c.1138G>A (p.G380R) | P | Achondroplasia | - | Postnatal blood | - |
| 70 | Abnormal Ultrasound | | Skeletal | 32.9 | 26 | 30 | *FGFR3* | c.1138G>A (p.G380R) | P | Achondroplasia | - | Postnatal blood | Full term delivery |
| 71 | Abnormal Ultrasound | | Skeletal | 36.7 | 23 | 24 | *COL1A1* | c.4287C>A (p.Y1429*) | P | Osteogenesis imperfecta | - | - | - |
| 72 | Abnormal Ultrasound | | Skeletal | 30.9 | 38 | 43 | *FGFR3* | c.1138G>A (p.G380R) | P | Achondroplasia | - | - | - |
| 73 | Abnormal Ultrasound | | Skeletal | 26.4 | 31 | 31 | *COL1A1* | c.2210G>A (p.G737D) | P | Osteogenesis imperfecta | - | Postnatal clinical exam | Postnatal demise |
| 74 | Abnormal Ultrasound | | Skeletal | 34.4 | 36 | 44 | *FGFR3* | c.1620C>A (p.N540K) | P | Hypochondroplasia | - | - | - |
| 75 | Abnormal Ultrasound | | Skeletal | 29.9 | 25 | 25 | *FGFR2* | c.758C>G (p.P253R) | P | Apert syndrome | - | - | - |
| 76 | Abnormal Ultrasound | | Skeletal | 32.7 | 32 | 36 | *FGFR3* | c.1138G>A (p.G380R) | P | Achondroplasia | - | - | Full term delivery |
| 77 | Abnormal Ultrasound | | Skeletal | 28.3 | 30 | 46 | *FGFR3* | c.742C>T(p.R248C) | P | Thanatophoric dysplasia | - | Postnatal clinical exam | Preterm delivery |
| 78 | Abnormal Ultrasound | | Skeletal | 31.4 | 36 | 38 | *FGFR3* | c.1138G>A (p.G380R) | P | Achondroplasia | - | Postnatal blood | Full term delivery |
| 79 | Abnormal Ultrasound | | Skeletal | 25.4 | 30 | 32 | *FGFR3* | c.1948A>G (p.K650E ) | P | Thanatophoric dysplasia | - | - | Postnatal demise |
| 80 | Abnormal Ultrasound | | Skeletal | 25.5 | 32 | 36 | *NIPBL* | c.7574_7587del(p.C2525Ffs*32) | LP | Cornelia de Lange | - | - | Elective termination |
| 81 | Abnormal Ultrasound | | Facial/Cranial | 30.9 | 29 | 21 | *FGFR2* | c.758C>T (p.P253L) | LP | Craniosynostosis | Paternal | Postnatal clinical exam | Full term delivery |
| 82 | Abnormal Ultrasound | | Facial/Cranial | 28 | 33 | 40 | *FGFR2* | c.758C>G (p.P253R) | P | Apert syndrome | - | - | - |
| 83 | Abnormal Ultrasound | | Facial/Cranial | 25.7 | 30 | 29 | *FGFR2* | c.758C>G (p.P253R) | P | Apert syndrome | - | - | - |
| 84 | Abnormal Ultrasound | | Facial/Cranial | 23.1 | 35 | 37 | *FGFR2* | c.940-2A>G | P | Craniosynostosis | - | - | - |
| 85 | Abnormal Ultrasound | | Facial/Cranial | 26.3 | 39 | 41 | *FGFR2* | c.755C>G (p.S252W) | P | Apert syndrome | - | Postnatal blood | - |
| 86 | Abnormal Ultrasound | | Facial/Cranial | 32.4 | 26 | 35 | *FGFR2* | c.870G>T (p.W290C) | P | Pfeiffer syndrome |  | Postnatal blood | Full term delivery |
| 87 | Abnormal Ultrasound | | Lymphatic | 11.4 | 26 | 27 | *PTPN11* | c.922A>G (p.N308D) | P | Noonan spectrum disorder | Maternal | Chorionic villus sampling | - |
| 88 | Abnormal Ultrasound | | Lymphatic | 23.7 | 31 | 31 | *PTPN11* | c.188A>G (p.Y63C) | P | Noonan spectrum disorder | Maternal | - | - |
| 89 | Abnormal Ultrasound | | Lymphatic | 12 | 39 | 41 | *COL1A2* | c.738+1G>C | LP | Osteogenesis imperfecta | Maternal | - | Full term delivery |
| 90 | Abnormal Ultrasound | | Lymphatic | 14.3 | 37 |  | *SOS1* | c.806T>C(p.M269T) | P | Noonan spectrum disorder | Maternal | Amniocentesis, Parental blood | - |
| 91 | Abnormal Ultrasound | | Lymphatic | 16.6 | 31 | 26 | *SOS1* | c.805A>G(p.M269V) | LP | Noonan spectrum disorder | Maternal | Parental blood | - |
| 92 | Abnormal Ultrasound | | Lymphatic | 21.1 | 32 | 32 | *RIT1* | c.229G>A (p.A77T) | P | Noonan spectrum disorder | - | Amniocentesis | Elective termination |
| 93 | Abnormal Ultrasound | | Lymphatic | 20.4 | 35 | 28 | *RIT1* | c.270G>T(p.M90I) | P | Noonan spectrum disorder | - | Postnatal blood | Postnatal demise |
| 94 | Abnormal Ultrasound | | Lymphatic | 24 | 26 | 34 | *KRAS* | c.458A>T (p.D153V) | P | Noonan spectrum disorder | - | Postnatal blood | Full term delivery |
| 95 | Abnormal Ultrasound | | Lymphatic | 12.4 | 33 | 35 | *PTPN11* | c.188A>G (p.Y63C) | P | Noonan spectrum disorder | - | Chorionic villus sampling | Full term delivery |
| 96 | Abnormal Ultrasound | | Lymphatic | 21.1 | 30 | 55 | *PTPN11* | c.215C>G (p. p.A72G) | P | Noonan spectrum disorder | - | - | - |
| 97 | Abnormal Ultrasound | | Lymphatic | 18.1 | 32 | 38 | *BRAF* | c.1390G>A (p.G464R) | P | Noonan spectrum disorder | - | Postnatal blood | - |
| 98 | Abnormal Ultrasound | | Lymphatic | 13.6 | 23 | 23 | *NIPBL* | c.611-1G>C | P | Cornelia de Lange | - | - | - |
| 99 | Abnormal Ultrasound | | Lymphatic | 24.9 | 19 | 21 | *PTPN11* | c.184T>G (p.Y62D) | P | Noonan spectrum disorder | - | Products of conception | - |
| 100 | Abnormal Ultrasound | | Lymphatic | 14 | 34 | 29 | *RIT1* | c.246T>G (p.F82L) | P | Noonan spectrum disorder | - | - | - |
| 101 | Abnormal Ultrasound | | Lymphatic | 22.3 | 34 | 36 | *PTPN11* | , c.227A>T (p.E76V) | P | Noonan spectrum disorder | - | - | - |
| 102 | Abnormal Ultrasound | | Lymphatic | 27.7 | 44 | 49 | *SHOC2* | c.4A>G (p.S2G) | P | Noonan spectrum disorder | - | Postnatal blood | - |
| 103 | Abnormal Ultrasound | | Lymphatic | 27.4 | 25 | 34 | *RAF1* | c.770C>T (p.S257L) | P | Noonan spectrum disorder | - | Postnatal blood | - |
| 104 | Abnormal Ultrasound | | Lymphatic | 12.6 | 29 | 30 | *BRAF* | c.1455G>T (p.L485F) | P | Noonan spectrum disorder | - | Amniocentesis | - |
| 105 | Abnormal Ultrasound | | Lymphatic | 11.9 | 33 | 31 | *PTPN11* | c.182A>T (p.D61V) | P | Noonan spectrum disorder | - | - | - |
| 106 | Abnormal Ultrasound | | Lymphatic | 26.1 | 29 | 32 | *RAF1* | c.770C>T (p.S257L) | P | Noonan spectrum disorder | - | - | - |
| 107 | Abnormal Ultrasound | | Cardiac | 31.3 | 25 | 26 | *TSC2* | c.5160+2T>C | P | Tuberous sclerosis | - | - | - |
| 108 | Abnormal Ultrasound | | Cardiac | 28.3 | 31 | 31 | *TSC2* | c.2220+1G>T | P | Tuberous sclerosis | - | - | Full term delivery |
| 109 | Abnormal Ultrasound | | Cardiac | 31.4 | 32 | 38 | *TSC2* | c.4936delG (p.V1646Cfs*26) | P | Tuberous sclerosis | - | - | Full term delivery |
| 110 | Abnormal Ultrasound | | Cardiac | 26.6 | 34 | 28 | *NIPBL* | c.4787_4791delTCAGT (p.F1596*) | P | Cornelia de Lange | - | - | Preterm delivery |
| 111 | Abnormal Ultrasound | | Other | 25.4 | 35 | 40 | *NIPBL* | c.1435C>T (p.R479*) | P | Cornelia de Lange | - | Products of conception | - |
| 112 | Abnormal Ultrasound | | Other | 25.1 | 31 | 29 | *FGFR2* | c.870G>T (p.W290C) | P | Pfeiffer syndrome | - | - | Full term delivery |
| 113 | Abnormal Ultrasound | | Other | 25 | 36 | 36 | *FGFR3* | c.1138G>A (p.G380R) | P | Achondroplasia | - | Postnatal clinical exam | Full term delivery |
| 114 | Abnormal Ultrasound | | Other | 24.3 | 22 | 22 | *FGFR2* | c.755C>G (p.S252W) | P | Apert syndrome | - | Postnatal clinical exam | Full term delivery |
| 115 | Abnormal Ultrasound | | Other | 12.7 | 24 | 24 | *COL1A2* | c.3007G>C (p.G1003R) | P | Osteogenesis imperfecta | - | - | Elective termination |
| 116 | Abnormal Ultrasound | | Other | 22.4 | 29 | 30 | *PTPN11* | c.188A>G (p.Y63C) | P | Noonan spectrum disorder | - | - | - |
| 117 | Abnormal Ultrasound | | Not Available | 29.7 | 22 | 27 | *FGFR3* | c.746C>G (p.P249C) | P | Thanatophoric dysplasia | - | Postnatal blood | Postnatal demise |
| 118 | Abnormal Ultrasound | | Not Available | 20.3 | 33 | 36 | *FGFR3* | c.742C>T (p.R248C) | P | Thanatophoric dysplasia | - | Postnatal blood | Postnatal demise |
| 119 | Abnormal Ultrasound | | Not Available | 24.9 | 17 | 18 | *COL1A2* | c.3107G>A (p.G1036D) | P | Osteogenesis imperfecta | - | Postnatal clinical exam | Stillbirth |
| 120 | Screening – Advanced paternal age | |  | 11.4 | 36 | 46 | *BRAF* | c.722C>T(p.T241M) | P | Noonan spectrum disorder | Maternal | - | - |
| 121 | Screening – Advanced maternal age | |  | 12.7 | 36 | 33 | *COL1A2* | c.96+2T>G | LP | COL1A2 related disorders | Maternal | - | Full term delivery |
| 122 | Screening- Advanced maternal age/ Advanced paternal age | |  | 10.3 | 36 | 60 | *CDKL5* | c.118G>A (p.A40T) | LP | X-linked dominant early infantile epileptic encephalopathy-2 | - | Amniocentesis | - |
| 123 | No indication provided | |  | 11.6 | 27 | 28 | *COL1A1* | c.2641G>A (p.G881S) | LP | Osteogenesis imperfecta | Maternal | Amniocentesis | Full term delivery |
| 124 | No indication provided | |  | 9.6 | 37 | 36 | *SYNGAP1* | c.67+2_67+18del17 | LP | Autosomal dominant mental retardation-5 | - | - | - |
| 125 | No indication provided | |  | 10.2 | 35 | 28 | *CBL* | c.1111T>A (p.Y371N) | P | Noonan spectrum disorder | - | Chorionic villus sampling | Elective termination |
| * P/LP Pathogenic or Likely Pathogenic | | | | | | | | | | |  |  |  |
